# Hot Topics in Implant-Based Breast Reconstruction

**DOI:** 10.3390/jcm15010263

**Published:** 2025-12-29

**Authors:** Thomas J. Sorenson, Carter J. Boyd, Nolan S. Karp

**Affiliations:** Hansjorg Wyss Department of Plastic Surgery, NYU-Langone Health, New York, NY 10016, USA; thomas.sorenson@nyulangone.org (T.J.S.);

**Keywords:** breast implant, breast reconstruction, nipple-sparing mastectomy, prepectoral, neurotization, breast implant-associated anaplastic large cell lymphoma, BII, acellular dermal matrix, mesh

## Abstract

Implant-based breast reconstruction (IBBR) remains the most common form of post-mastectomy reconstruction worldwide, offering patients a reliable and accessible option to restore breast contour. Advances in surgical technique, biomaterials, and implant technology have driven rapid evolution in the field, with the dual goals of improving aesthetic outcomes and minimizing patient morbidity. The prepectoral plane has been popularized due to the eliminated risk of animation deformity and reduced postoperative pain. Some concerns remain regarding mastectomy flap thickness and long-term oncologic and aesthetic outcomes. Concurrently, nipple-sparing mastectomy has improved aesthetic results and enabled surgeons to move beyond just restoring breast form and improve functional recovery as well, as demonstrated by surgical efforts aimed at restoring nipple–areolar complex (NAC) sensation. Adjunctive use of biologic matrices and synthetic meshes has broadened reconstructive options, while next-generation implants seek to further enhance outcomes. Balanced against these innovations are important oncologic and systemic safety concerns, including breast implant-related cancers and the ongoing debate over breast implant illness (BII). This review highlights eight current “hot topics” in implant-based breast reconstruction: (1) prepectoral reconstruction, (2) nipple-sparing mastectomy, (3) oncoplastic techniques, (4) nipple–areolar complex (NAC) neurotization, (5) biologic matrices and synthetic meshes, (6) next-generation implants, (7) optimizing aesthetic outcomes, and (8) implant-associated cancer and systemic concerns. Together, these areas define the current landscape of innovation, controversy, and future directions in implant-based reconstruction.

## 1. Introduction

Implant-based breast reconstruction (IBBR) remains the most performed method of post-mastectomy reconstruction worldwide. Over the past decades, advances in surgical technique, biomaterials, and implant technology have driven a paradigm shift in how these procedures are performed. Surgeons have moved away from traditional subpectoral placement toward prepectoral reconstruction, enabled by improved mastectomy flap viability and the widespread use of acellular dermal matrices (ADMs) and synthetic meshes. At the same time, increasing adoption of nipple-sparing mastectomy (NSM) has transformed both the aesthetic goals and the technical challenges of reconstruction. While restoring breast form has always been central, contemporary practice also prioritizes function, sensation, and patient-reported satisfaction. Emerging efforts at nipple–areolar complex (NAC) neurotization reflect this trend, as do adjunctive strategies such as autologous fat grafting and 3D-based aesthetic analysis. Parallel to these surgeon-driven innovations, industry advances in implant design also seek to improve both safety and outcomes.

Nevertheless, controversy persists. Cancer recurrences after NSM, breast implant-associated anaplastic large cell lymphoma (BIA-ALCL), and the broader phenomenon of “breast implant illness” (BII) have raised new questions regarding oncologic safety and systemic risks of these breast reconstruction advances, and these concerns must be balanced against the clear benefits of implant-based approaches in appropriate candidates. This review highlights eight current “hot topics” in implant-based breast reconstruction: (1) prepectoral reconstruction, (2) nipple-sparing mastectomy, (3) oncoplastic techniques (4) nipple–areolar complex (NAC) neurotization, (5) biologic matrices and synthetic meshes, (6) next-generation implants, (7) optimizing aesthetic outcomes, and (8) implant-associated cancer and systemic concerns. Together, these areas define the current landscape of innovation, controversy, and future directions in implant-based reconstruction.

## 2. Prepectoral Breast Reconstruction

Prepectoral breast reconstruction has undergone a remarkable evolution in a relatively short period, reflecting both improvements in oncologic breast surgery and sophisticated refinements in implant-based reconstruction [1]. Historically, subpectoral implant placement was considered the gold standard because muscular coverage was believed to reduce implant visibility, protect against capsular contracture, and lower the risk of extrusion. However, as mastectomy techniques improved and skin flap perfusion became more reliable, the question emerged as to whether the pectoralis major muscle, whose primary function is unrelated to breast reconstruction, needed to be sacrificed at all.

Prepectoral reconstruction avoids elevation of the pectoralis major muscle, eliminating postoperative muscle spasm and reducing pain [2,3,4]. Multiple studies now demonstrate that patients experience an earlier return to baseline arm function, fewer activity limitations, and quicker resumption of normal daily life [5,6,7,8,9,10]. As enhanced recovery after surgery (ERAS) protocols become widespread, prepectoral placement aligns well with patient-centered goals, decreasing opioid requirements and facilitating outpatient pathways.

Another widely cited advantage is the absence of animation deformity. In subpectoral reconstruction, contraction of the pectoralis muscle can distort the implant, creating visible tectonic shifts that are distressing to many patients, particularly those who are athletic or physically active [11]. Prepectoral placement fully eliminates this problem, representing a meaningful improvement in aesthetic stability across dynamic positions.

Mastectomy flap viability remains the rate-limiting step. Prepectoral reconstruction relies on robust, well-perfused soft tissue to provide long-term coverage of the device. In additional to clinical exam, surgeons now have access to intraoperative perfusion imaging, like indocyanine green (ICG) angiography, to guide real-time decisions about flap viability [12]. When flaps appear marginal, surgeons may partially or completely abandon prepectoral placement in favor of a dual-plane or subpectoral approach. This adaptability is essential, as compromised flaps increase the risk of mastectomy flap necrosis, infection, and early implant loss.

Large contemporary analyses and multiple systematic reviews demonstrate that overall complication rates are comparable between prepectoral and subpectoral reconstruction, provided appropriate patient selection [2,13,14,15]. Particularly important is the growing evidence on patient-reported outcomes, showing equal or improved satisfaction with physical well-being of the chest and aesthetic appearance in prepectoral groups [9,10,16,17,18]. These data reinforce that the shift toward prepectoral placement is not only technically feasible but also valued by patients.

Despite these advances, prepectoral reconstruction introduces unique challenges. The lack of muscular coverage makes implants more palpable, particularly in thin patients, and less durable against skin flap insults [19,20,21]. Rippling, implant edge visibility, and upper pole contour irregularities may require secondary fat grafting. Seroma formation is another frequently debated issue. Some series suggest higher seroma rates in prepectoral reconstruction, especially when large ADM sheets are used, though data remain mixed. Recent work highlights the importance of controlling expander fill volumes and minimizing shear forces on the mastectomy flaps to prevent seroma and implant displacement [22,23].

Long-term durability is another area of active investigation. Whether prepectoral implants experience higher rates of bottoming out, lateral migration, or capsular contracture compared with subpectoral placement remains uncertain. Long-term longitudinal data will better answer these questions. Nevertheless, as surgeons refine flap assessment, optimize pocket control, and improve mesh-implant constructs, prepectoral reconstruction is poised to remain a foundational technique in modern implant-based reconstruction.

## 3. Nipple-Sparing Mastectomy

Nipple-sparing mastectomy (NSM) has arguably been one of the most transformative developments in contemporary breast cancer surgery [24,25,26,27]. Its rise parallels the shifting focus from merely removing cancer to incorporating aesthetics, body image, and psychosocial recovery into surgical planning [28]. From a reconstructive standpoint, NSM offers unparalleled advantages, preserving the native nipple position, contour, and pigmentation. Patients consistently report higher satisfaction with breast appearance, body image, and sexual well-being compared with traditional mastectomy [29]. The technique, however, introduces a new set of challenges.

The oncologic safety of NSM has been the subject of substantial investigation [30,31,32]. Early hesitations centered on the possibility of residual glandular tissue beneath the nipple–areolar complex (NAC) and the consequent risk of local recurrence. However, multiple long-term series and multi-institutional studies demonstrate that in carefully selected patients, NSM carries oncologic outcomes equivalent to skin-sparing or total mastectomy [33,34,35]. Patient selection remains critical, with tumor-to-nipple distance, retroareolar involvement, and intraoperative frozen sections playing key roles in decision making (Table 1). In prophylactic settings, NSM is widely considered safe, although rare reports of new primaries within the preserved NAC have been described [36].

NSM introduces specific technical challenges for breast reconstruction. Nipple ischemia and partial or total NAC necrosis remain important complications, with risk factors including periareolar incisions, smoking, prior radiation, diabetes, elevated BMI, and thin or compromised flaps [20]. Adoption of inframammary or lateral incisions, meticulous tissue handling, and reliance on ICG angiography have dramatically reduced complication rates [37,38]. Yet even with optimal technique, partial NAC necrosis remains a possibility, emphasizing the need for thoughtful patient counseling.

NSM has also altered the aesthetics of implant-based reconstruction. Because the NAC is preserved, the reconstructed breast must achieve harmony in shape, projection, and upper pole contour. This often synergizes with prepectoral reconstruction and ADM or mesh reinforcement, which allow for fine control of pocket dynamics and projection [23,39]. Incision placement is also crucial; lateral radial and inframammary incisions provide the best balance of access and aesthetic concealment.

Increasingly, NSM is also connected to functional restoration, particularly through NAC neurotization [40]. Patients often express greater disappointment with loss of sensation than with loss of the breast itself. NSM preserves the external anatomy, but without neurotization, the nipple remains insensate. As NSM becomes standard, functional restoration becomes an increasingly important adjunct. Overall, NSM represents a convergence of oncologic safety, aesthetic outcomes, and functional advancements. As surgical and reconstructive techniques continue to improve, NSM will remain central to modern breast reconstruction strategies.

## 4. Oncoplastic Techniques

Oncoplastic techniques have broadened reconstructive possibilities for patients undergoing mastectomy or treatment after breast conserving therapy (BCT), bridging the gap between aesthetic breast surgery principles and oncologic safety [41,42,43,44,45]. Among these techniques, the Wise-pattern mastectomy (with or without the inferior dermal flap) and implant-based reconstruction after lumpectomy and radiation represent two scenarios with high aesthetic expectations and notable reconstructive challenges.

Wise-pattern mastectomy allows for oncologic resection in patients with macromastia or significant breast ptosis by combining a skin-reducing pattern with predictable control of the skin envelope [46,47]. This facilitates creation of an ideal breast footprint and nipple position while eliminating redundant tissue that would otherwise compromise implant positioning. For patients with large or ptotic breasts, a traditional skin-sparing mastectomy may leave an oversized, poorly draping envelope that increases the risk of malposition, bottoming out, and poor implant aesthetics.

The Wise-pattern approach enables immediate placement of an appropriately sized implant or tissue expander in a tailored envelope shaped to match the final breast form [46]. However, the technique introduces unique reconstructive risks. The T-point, the intersection of vertical and inframammary incisions, remains a well-recognized area of vascular compromise. Partial skin necrosis at this site can expose the implant, require debridement, or disrupt pocket integrity. The Wise-pattern approach uniquely allows for the creation of an inferior dermal flap (“autoderm”) that can be used as a vascularized tissue layer between the implant and the mastectomy skin flap, which is especially useful at the area of the T-point [48,49]. Even with this autoderm flap, the use of prepectoral implants in Wise-pattern cases requires careful flap perfusion and often necessitates protective measures such as staged reconstruction, mesh-supported pockets, or intraoperative downsizing of the implant.

Despite these challenges, aesthetic outcomes can be excellent when flap viability is preserved. The Wise pattern allows for precise repositioning of the nipple–areolar complex, predictable control of lower pole projection, and improved symmetry for bilateral cases. Patients with significant ptosis often prefer the postoperative breast shape achieved with this pattern, as it avoids the bottomed-out appearance that may occur without skin reduction. In certain cases, NSM can be performed via the Wise pattern with the NAC being perfused by a dermal flap [50]. If this is not possible but saving the NAC is oncologically safe, free nipple grafting has also been used successfully to provide an aesthetic and oncologically safe result [51,52]. Future directions in this area include refined perfusion-based algorithms, hybrid reconstruction strategies (implant plus fat grafting), and selective use of biosynthetic mesh to decrease tension on the T-junction and improve durability of lower pole support.

Reconstruction after lumpectomy and radiation represents a distinct subset of implant-based reconstruction with biomechanical and aesthetic considerations that differ from immediate post-mastectomy reconstruction [53,54,55]. Radiation induces fibrosis, volume loss, contour deformity, and skin tightening that can create a challenging environment for later implant placement. Increasing numbers of patients treated initially with lumpectomy and radiation later desire breast reshaping or develop deformities substantial enough to warrant conversion to mastectomy with implant-based reconstruction.

For patients undergoing mastectomy after BCT, implant-based reconstruction must account for the decreased elasticity, compromised microvasculature, and higher infection and capsular contracture rates associated with prior radiation. Subpectoral placement of the implant has been preferred, sometimes combined with mesh to achieve pocket control, to offer better soft-tissue protection [54]. However, oncologic safety of either plane is debated, with some studies showing subpectoral as safe and some showing prepectoral as safer [56,57].

When deformity occurs after lumpectomy without mastectomy, options for implant-based contour correction include small-volume implants, with occasional combined fat-implant approaches. Fat grafting remains a central tool in radiated tissue, serving both an aesthetic and regenerative role by improving pliability and soft-tissue thickness, but patients may require multiple sessions for optimal correction. Outcomes after implant-based reconstruction in the post-BCT setting are generally less predictable than those after mastectomy in non-irradiated patients. Capsular contracture, especially high grade, is a known risk, often necessitating secondary reconstruction or conversion to autologous tissue [53,54]. For patients who strongly prefer implants, careful preoperative counseling and shared decision making are critical to establishing realistic expectations.

Despite these challenges, selected patients can achieve good aesthetic and functional outcomes, particularly when reconstruction is staged and supported by adjunctive fat grafting or mesh reinforcement [58]. Ongoing research into radiation-mitigating therapies and expanded indications for neurotization and biosynthetic support materials may further improve implant outcomes in this historically high-risk population.

## 5. Nipple–Areolar Complex (NAC) Neurotization

**Figure 1:**
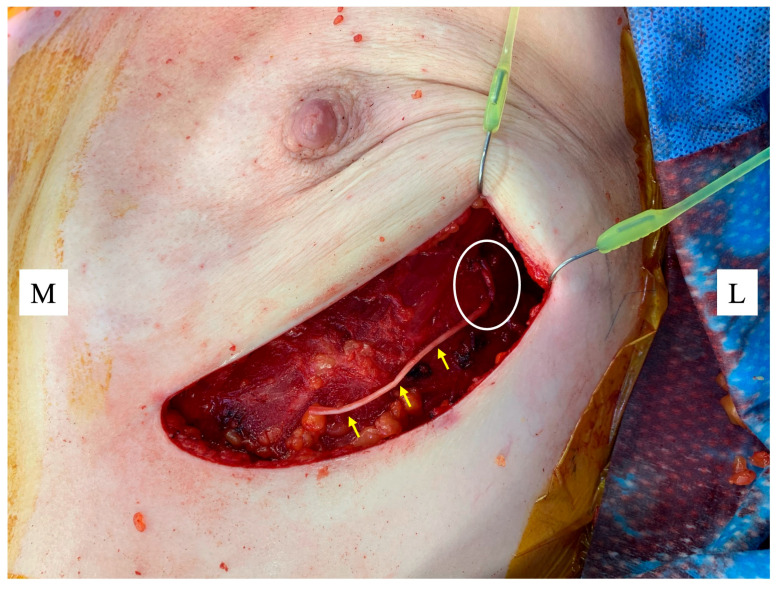
Nerve allograft (yellow arrows) coapted to a lateral intercostal nerve as it emerges from the chest wall (white circle) ready for suturing to the underside of the nipple areolar complex (NAC). Figure key: M, medial; L, lateral.

Restoration of NAC sensation is a frontier that reflects the maturation of IBBR from purely cosmetic restoration to comprehensive functional rehabilitation. Sensory loss can negatively influence sexual function, embodiment, temperature perception, and overall quality of life [59,60]. Traditional mastectomy severs intercostal nerve branches that supply the breast skin and NAC [61,62]. Without intentional reconstruction of this pathway, sensory recovery is limited and unpredictable. NAC neurotization aims to address this by re-establishing innervation through coaptation of preserved intercostal nerve branches to the undersurface of the NAC using nerve allografts or autografts [63] (Figure 1).

Early outcomes from multiple centers report encouraging results. Many patients experience an earlier and more robust return of light-touch and protective sensation compared with controls who did not undergo neurotization [64,65,66]. These results appear durable. In some studies, patients undergoing neurotization report improved scores in patient-reported outcome measures compared to controls [67,68].

However, significant challenges remain, including technical standardization, cost and operative time, and the need for high-quality evidence [69]. Different surgeons use different donor nerves, graft types (allograft vs. autograft), tunnel pathways, and coaptation techniques. Without standardization, comparative effectiveness research remains difficult. Neurotization adds operative complexity and expense, which is an important consideration in a procedure already scrutinized for cost effectiveness. Despite encouraging early results, the current evidence supporting NAC neurotization is limited by heterogeneous surgical techniques, variable sensory testing methodologies, and a predominance of non-randomized study designs. Most published data derive from retrospective series or prospective cohorts, and randomized controlled trials are lacking. As such, NAC neurotization should currently be considered an emerging adjunct rather than a standard component of implant-based reconstruction. Future prospective, ideally randomized, studies with standardized neurotization techniques and validated sensory outcome measures are necessary to define its true clinical benefit and cost effectiveness.

Despite these limitations, NAC neurotization aligns strongly with patient priorities. As the concept of oncoplastic reconstruction continues to evolve, NAC neurotization represents an important step toward expanding the goals of IBBR from purely aesthetic restoration to functional recovery.

## 6. Optimizing Aesthetic Outcomes

While IBBR reliably restores breast form, optimizing aesthetic outcomes remains a central focus of contemporary practice. Increasingly, patients are expecting a highly natural-appearing result that closely mimics (or even improves upon) their original, natural breasts. Thus, aesthetic success depends not only on implant placement but also on careful management of mastectomy flaps, adjunctive procedures, and the establishment of long-term symmetry. Adjunctive fat grafting has become an essential tool for refining contour, correcting rippling, and improving soft tissue coverage [70]. Beyond aesthetics, fat grafting may also enhance flap vascularity and reduce radiation-induced fibrosis, though concerns about oncologic safety continue to be studied [71]. This is especially important in revision reconstruction, where radiation or weight fluctuations have distorted normal anatomy. Nipple reconstruction may also be desired after skin-sparing mastectomy (SSM).

Pocket selection and control also play critical roles. The use of the prepectoral plane can improve upper pole contour and eliminate animation deformity but requires well-vascularized mastectomy flaps. In addition to lateral pocket plication sutures, mesh-implant constructs can also be used to secure the implant in the breast pocket and create a stable implant position [23]. These mesh-implant constructs can also be used in revision procedures to restore symmetry after radiation or weight loss-induced changes. Patient-reported outcomes are equally critical. Instruments such as the BREAST-Q have demonstrated that aesthetic success is strongly tied to patient satisfaction, body image, and psychosocial well-being. These measures highlight that even technically “ideal” results must be interpreted through the lens of patient perception.

Nipple reconstruction, when needed, contributes to aesthetic completeness. Even in an era where NSM predominates, nipple reconstruction remains relevant for patients undergoing skin-sparing mastectomy. Techniques range from local flap reconstruction to tattoo-only approaches, each offering different balances of projection, longevity, and complexity, and can be performed in a delayed or immediate fashion [72].

Managing patient expectations is fundamental. Revision strategies remain common even in supposedly “one-stage DTI”-based breast reconstruction, and capsular contracture, asymmetry, malposition, and rippling may necessitate secondary procedures. Understanding risk factors, especially the use of radiation, is essential for setting patient expectations regarding the anticipated result. Ultimately, optimizing aesthetic outcomes in IBBR requires a multimodal and thoughtful methodical approach to achieve durable, natural-appearing results, and surgeons must counsel patients that achieving symmetry and natural appearance frequently requires staged interventions.

## 7. Biologic Matrices and Synthetic Meshes

The introduction of biologic matrices and synthetic meshes has transformed IBBR by expanding reconstructive options and improving implant support [22] (Table 2). Initially popularized in subpectoral reconstructions, acellular dermal matrices (ADMs) provided inferolateral coverage, pocket control, and improved aesthetic contour, though they are not explicitly approved by the FDA for use in the breast [73,74,75]. Their use has since extended to prepectoral reconstruction, where an ADM is frequently used to provide a layer of reinforcement between the implant and mastectomy flaps, helping to reduce complications and improve cosmesis [76,77]. Despite these benefits, ADM use remains controversial. Studies have demonstrated associations with higher rates of seroma, infection, and red breast syndrome, though findings are inconsistent across institutions [78,79]. Cost also poses a significant barrier, as ADMs can account for a substantial portion of reconstructive expenses, raising questions about cost effectiveness in the absence of clear long-term outcome advantages [80]. While some studies demonstrate improved aesthetic and patient-reported outcomes with ADMs, others show minimal difference when compared with no-ADM reconstruction. This heterogeneity reflects differences in patient selection, flap quality, implant plane, and ADM type.

As cost and complication concerns surrounding ADMs grew, synthetic and biosynthetic meshes emerged as alternatives. These materials, including P4HB (GalaFLEX), TIGR mesh, PDO (Durasorb), and other long-term resorbable scaffolds, offer temporary support that is gradually replaced by native tissue [81,82]. Early evidence suggests comparable complication rates to ADMs with lower costs and potentially fewer seromas [83,84]. Biosynthetic meshes are especially attractive for prepectoral DTI reconstruction, where they provide a semi-rigid anterior layer that prevents folding, supports projection, and reduces implant motion. The long-term performance of synthetic scaffolds remains under study. As these materials resorb, the durability of the collagenous capsule they leave behind becomes critical. Emerging data, including from studies of P4HB constructs in prepectoral DTI reconstruction, suggest promising stability and low complication rates [48].

Ultimately, matrix and mesh selection should be individualized. The optimal reinforcement strategy depends on flap thickness, patient comorbidities, radiation history, implant plane, and surgeon expertise.

## 8. Next-Generation Implants

Advances in implant technology continue to shape the landscape of IBBR (Table 3). Historically, concerns about implant rupture, capsular contracture, and textured implant safety have driven innovation toward safer and more durable devices, and current development has focused on improving biocompatibility and enhancing patient satisfaction. One area of focus is breast implant surface design. Following the global recall of macrotextured implants due to their association with breast implant-associated anaplastic large cell lymphoma (BIA-ALCL), smooth and microtextured surfaces have become the standard [85,86]. Novel “nanotextured” implants were introduced to provide the theoretical benefits of texturing (reduced rotation and contracture) while minimizing oncologic risk, though long-term outcome data are still limited and initial enthusiasm has been tempered by reports of higher malposition and rotation rates [87,88]. They are also not yet approved by the Food and Drug Administration (FDA) for use in breast reconstruction.

The development of highly cohesive silicone gel implants, often referred to as “gummy bear” implants, has improved shape retention and reduced the risk of silicone bleed in the event of rupture [89]. More recently, lightweight implants incorporating microspheres or alternative fillers have been introduced to reduce implant weight by up to 30%, aiming to decrease strain on mastectomy flaps, lower pole descent, and chronic discomfort. Early reports suggest high levels of patient satisfaction, but robust long-term data remain lacking [90]. These devices are used widely for aesthetic breast augmentation but have been increasingly introduced for alloplastic breast reconstruction, with unpublished studies ongoing that investigate their use.

Beyond structural modifications, next-generation implants are being explored for integration with regenerative strategies. Research into bioactive coatings, antimicrobial surfaces, and drug-eluting technologies holds the potential to further reduce capsular contracture, infection, and other complications [91,92,93]. As innovation continues, the challenge remains to balance technological advances with rigorous long-term safety data.

## 9. Implant-Associated Cancers and Systemic Concerns

The safety of breast implants has come under renewed scrutiny with the recognition of rare but serious implant-associated malignancies and the increasing attention to systemic symptomatology reported by some patients. Breast implant-associated anaplastic large cell lymphoma (BIA-ALCL) is a rare T-cell lymphoma that develops in the capsule surrounding textured implants, most commonly presenting as late-onset periprosthetic seroma or, less frequently, as a capsular mass [85,94]. After diagnosis confirmation with biopsy of the seroma fluid or mass, management typically involves total capsulectomy and implant removal, with most patients achieving durable remission when diagnosed early. These findings have led to global recalls of macrotextured implants and changes in regulatory guidance [86].

More recently, breast implant-associated squamous cell carcinoma (BIA-SCC) has been described, though fewer than 20 cases have been reported worldwide [95,96,97]. Importantly, and different from BIA-ALCL, BIA-SCC has a very aggressive clinical course, which should prompt heightened clinical concern. Unlike BIA-ALCL, which is strongly linked to textured implants, BIA-SCC has been documented in association with both smooth and textured implants, as well as in both aesthetic and reconstructive populations, and reported latency periods are long, often 10 or more years after initial implantation [97]. Clinically, BIA-SCC can present like BIA-ALCL, with progressive pain, breast swelling, and/or capsular contracture. Unlike BIA-ALCL, which frequently presents with a late periprosthetic effusion, BIA-SCC is more often associated with a solid tumor component invading the capsule or adjacent tissues. Imaging typically reveals irregular capsular thickening or a mass abutting the implant, and diagnosis is confirmed through capsule or mass biopsy demonstrating keratinizing squamous cell carcinoma.

The true incidence of BIA-SCC remains unknown and is presumed to be extremely low, but it must be stressed that any new breast mass, persistent pain, or unexplained swelling in long-term implant patients warrants prompt imaging and biopsy. BIA-SCC appears to have a more aggressive natural history than BIA-ALCL, with multiple reported cases demonstrating chest wall invasion, lymph node metastases, or distant spread at presentation. This disease carries a very poor prognosis, with an estimated six-month mortality rate of about 10%, and one-year mortality rate of nearly 25% [96,97]. Treatment requires complete surgical resection including implant removal, total capsulectomy without spillage, and often mastectomy with resection of adjacent tissue, if present [95]. As awareness grows and molecular profiling advances, future studies may clarify the mechanisms underlying BIA-SCC and help stratify patient risk. For now, BIA-SCC underscores the importance of long-term follow-up in implant patients and reinforces the need for transparent, evidence-based counseling regarding both the benefits and rare but serious risks associated with alloplastic breast reconstruction.

In parallel, increasing numbers of patients have reported systemic symptoms attributed to their implants, collectively referred to as “breast implant illness” (BII), though this is much less common in patients undergoing breast reconstruction compared to patients undergoing breast augmentation [98]. While anecdotal reports suggest symptomatic improvement following explantation and anterior capsulectomy, there is no consensus on diagnostic criteria, pathophysiology, or causal linkage between implants and systemic disease [99]. Despite limited scientific evidence, patient advocacy has driven regulatory bodies and professional societies to acknowledge BII as an important clinical entity requiring empathetic, patient-centered counseling [100]. The recognition of implant-associated malignancies and the growing awareness of BII highlight the need for robust surveillance, transparent preoperative patient counseling, and continued research into implant safety.

## 10. Implant-Based Breast Reconstruction and Safety

Safety remains a fundamental consideration in all forms of breast reconstruction, particularly when foreign materials are introduced. Implant-based breast reconstruction carries device-specific risks, including infection, capsular contracture, implant malposition or failure, and rare but serious implant-associated malignancies, which necessitate long-term surveillance and transparent patient counseling. The adjunctive use of acellular dermal matrices and biosynthetic meshes introduces additional considerations related to seroma formation, infection risk, and cost, though current evidence suggests comparable overall complication rates when these materials are used selectively and with appropriate technique. Importantly, safety considerations in implant-based reconstruction must be interpreted in context.

Autologous reconstruction, including DIEP and other free-flap techniques, avoids implant-related risks but introduces distinct safety trade-offs, such as donor-site morbidity, longer operative times, higher physiologic demand, and the potential for partial or total flap loss. Although randomized controlled trials comparing implant-based and autologous breast reconstruction across all outcome domains would be ideal, such studies remain limited due to challenges in patient preference, surgeon expertise, and ethical equipoise. As a result, much of the existing comparative literature consists of observational studies and patient-reported outcome analyses. Consequently, no reconstructive modality can be considered universally “safer,” and optimal reconstruction requires individualized risk stratification that accounts for patient comorbidities, oncologic treatment, anatomic considerations, and patient priorities. Framing safety as reconstructive method specific rather than hierarchical allows for balanced, patient-centered decision making in contemporary breast reconstruction.

## 11. Conclusions

Implant-based breast reconstruction continues to evolve rapidly, driven by advances in surgical technique, biomaterials, and implant design. Balanced against these innovations are important safety considerations, and the reconstructive surgeon must be careful to innovate while safeguarding patient trust and well-being. Future progress will depend on prospective, multicenter studies that establish standardized outcomes, long-term follow-up, and comparative effectiveness across reconstructive options. By addressing technical challenges, incorporating patient-centered metrics, and maintaining vigilance regarding safety, surgeons can continue to refine implant-based reconstruction to achieve durable, natural, and safe outcomes for patients.

## Figures and Tables

**Table 1:** Indications and contraindications for nipple-sparing mastectomy (NSM).

Category	Favorable Indications	Relative Contraindications	Absolute Contraindications
Oncologic factors	- Tumor ≥ 2 cm away from nipple on imaging/clinical exam - No evidence of nipple involvement on MRI or retroareolar biopsy - DCIS or invasive carcinoma confined to breast parenchyma	- Tumor-to-nipple distance < 2 cm - Multicentric disease without retroareolar involvement - Lobular histology (higher occult nipple involvement)	- Clinical or radiographic involvement of NAC - Positive retroareolar margin on frozen/permanent pathology - Inflammatory breast cancer
Patient factors	- Small to moderate breast size (<C cup) - None or minimal ptosis (Grade I–II) - Non-smoker - Normal BMI	- Large breast size (≥C cup) - Moderate ptosis (Grades II–III) - Remote smoking history - Prior breast surgery or irradiation	- Very large breast size - Severe ptosis that may compromise mastectomy skin flaps - Active smoking - Uncontrolled vascular comorbidities
Technical considerations	- Good mastectomy flap vascularity - Ability to use inframammary or lateral incision	- Need for periareolar or radial incision (higher ischemic risk)	- Inability to achieve oncologically safe resection with preserved NAC

Key: DCIS, ductal carcinoma in situ; BMI, body mass index.

**Table 2:** Comparison of acellular dermal matrix (ADM) vs. synthetic/biosynthetic meshes in implant-based breast reconstruction.

Feature	Acellular Dermal Matrix (ADM)	Synthetic/Biosynthetic Meshes
Source	Biological, human or porcine-derived extracellular matrix	Synthetic (e.g., polypropylene and polydioxanone) or biosynthetic (e.g., poly-4-hydroxybutyrate and TIGR mesh)
Tissue integration	Facilitates neovascularization and tissue incorporation	Gradual resorption with fibrous tissue replacement with variable integration depending on material
Pocket control/support	Provides soft-tissue reinforcement and implant coverage	Can provide more structural support and may be less pliable than ADM
Complications	Associated with higher rates of seroma, infection, and “red breast syndrome”	Generally lower seroma rates but long-term complication data still limited
Cost	High; barrier to widespread use	Lower; more cost effective in most settings

**Table 3:** Next-generation breast implants by brand and type.

Brand/Manufacturer	Implant Type	Key Features	Regulatory Status
Allergan Natrelle INSPIRA^®^ (AbbVie/Allergan Aesthetics)	Cohesive silicone gel implants (smooth, moderate/mid/high projection)	Wide range of profiles; highly cohesive gel (“gummy bear” type)	FDA and CE approved
Mentor MemoryGel^®^ Xtra (Johnson & Johnson)	Highly cohesive silicone gel	Increased projection and upper pole fullness; smooth shell	FDA and CE approved
Mentor MemoryShape^®^ (Johnson & Johnson)	Anatomically shaped, cohesive gel	“Tear-drop” profile for contour shaping	FDA approved
Sientra OPUS^®^ Silicone Gel Implants (Tiger Aesthetics)	Cohesive silicone gel (round and shaped)	U.S. only; smooth and microtextured surfaces	FDA approved (U.S. exclusive)
Motiva Ergonomix^®^ (Establishment Labs)	“Dynamic” silicone gel implant	Nanotextured “SilkSurface^®^”; designed to mimic natural movement	CE marked (not FDA approved for reconstruction)
Motiva Ergonomix2^®^ (Establishment Labs)	Next-gen with “BluSeal^®^” barrier and RFID chip	Enhanced safety features; real-time device identification	CE marked (not FDA approved for reconstruction)
Polytech Microthane^®^ (Polytech Health & Aesthetics)	Polyurethane-coated silicone implants	Lower capsular contracture rates; adherent surface	CE marked (not FDA approved in U.S.)
B-Lite^®^ (G&G Biotechnology)	Lightweight silicone gel + borosilicate microspheres	Up to 30% lighter; reduces implant weight burden	CE marked (not FDA approved in U.S.)
Sebbin Integrity^®^/Cristalline^®^ (Sebbin, France)	Cohesive gel implants (round and anatomical)	Customizable volumes and projections	CE marked (not FDA approved in U.S.)

## Data Availability

No new data were created or analyzed in this study. Data sharing is not applicable to this article.
